# Primary Rifampicin Mono-Resistant Extrapulmonary Tuberculosis of the Knee Without Pulmonary Involvement: One of the First Cases of Its Type in an Immunocompetent Male

**DOI:** 10.7759/cureus.47978

**Published:** 2023-10-30

**Authors:** Sankalp Yadav

**Affiliations:** 1 Medicine, Shri Madan Lal Khurana Chest Clinic, New Delhi, IND

**Keywords:** rifampicin mono-resistant tb, skeletal tuberculosis, histopathology, knee, cbnaat/ xpert/ rif assay, mtb (mycobacterium tuberculosis)

## Abstract

Tuberculosis presentation at sites other than the lungs is relatively infrequent, and isolated knee involvement without a pulmonary focus is exceptionally rare. Furthermore, there have been no reported cases of primary rifampicin mono-resistant extrapulmonary tuberculosis of the knee in males. In this case, a 24-year-old male patient presented with pain and swelling in his left knee after a fall five years ago. Given the absence of a history of tuberculosis, arriving at a diagnosis posed a significant challenge. However, the diagnosis was ultimately established through cartridge-based nucleic acid amplification tests and clinical link-up with radiometric techniques. Management was based on the latest national guidelines for anti-tuberculous treatment, which were tailored to his weight. To date, he has completed nine months of treatment with a significant improvement in his symptoms. This rare presentation emphasizes the need for a high degree of suspicion even in extrapulmonary tuberculosis cases.

## Introduction

Tuberculosis (TB) poses a grave public health concern in developing nations [1]. This ailment arises from an infection caused by acid-fast bacilli, specifically *Mycobacterium tuberculosis*, which belongs to the Mycobacteriaceae family [2]. According to the most recent reports from India, the incidence of multidrug-resistant (MDR) or rifampicin mono-resistant TB (RR-TB) patients in 2021 was approximately 119,000 (with a range of 93,000 to 145,000) [3]. Moreover, there was a notable 32% increment in the number of cases of MDR and RR-TB documented in 2022 compared to the previous year [3].

RR-TB is a form of TB in which the bacteria exhibit resistance to rifampicin [3]. There is a swift global escalation in the incidence of drug-resistant TB [3]. Typically, the disease primarily affects the lungs [4]. Nevertheless, sporadic instances of hematogenous dissemination, lymphatic spread, or localized disease development at extrapulmonary sites have been documented in the medical literature [4].

In this context, we present a case of a 28-year-old Indian male who came with swelling and pain in his left knee. He received a comprehensive clinical, radiometric, and laboratory evaluation, leading to a diagnosis of primary rifampicin mono-resistant extrapulmonary TB of the knee without pulmonary involvement, and was subsequently initiated on anti-tubercular treatment.

## Case presentation

In January 2023, a 24-year-old Indian male, who was unmarried and not diabetic, presented himself at the outpatient department. He reported experiencing pain and swelling in his left knee for a period of five years. The pain had a sudden onset, was persistent, did not radiate to other areas, intensified when walking (accompanied by a noticeable limp), and was relieved (for a period of three to four hours) upon taking over-the-counter medications. The swelling, on the other hand, had a gradual onset and had been progressively increasing over the past month. Notably, the patient did not exhibit symptoms such as fever, night sweats, cough, or a history of weight loss.

Approximately five years ago, the patient experienced a history of trauma resulting from a bike accident, which led to swelling in his left knee. During that time, the swelling occurred intermittently, prompting him to use over-the-counter medications and a crepe bandage for temporary relief. This self-administered treatment persisted for five years until he sought medical attention at the outpatient department, primarily because his swelling and pain had become continuous and were now significantly impeding his ability to walk.

In terms of his occupational history, he was employed at a cosmetic shop and had no prior history of TB or any close contacts with individuals affected by the disease. Additionally, it's important to note that he never smoked and was a staunch teetotaler. Besides, he had no prior history of migration, incarceration, or stints in shelters or relief camps.

**Figure 1:**
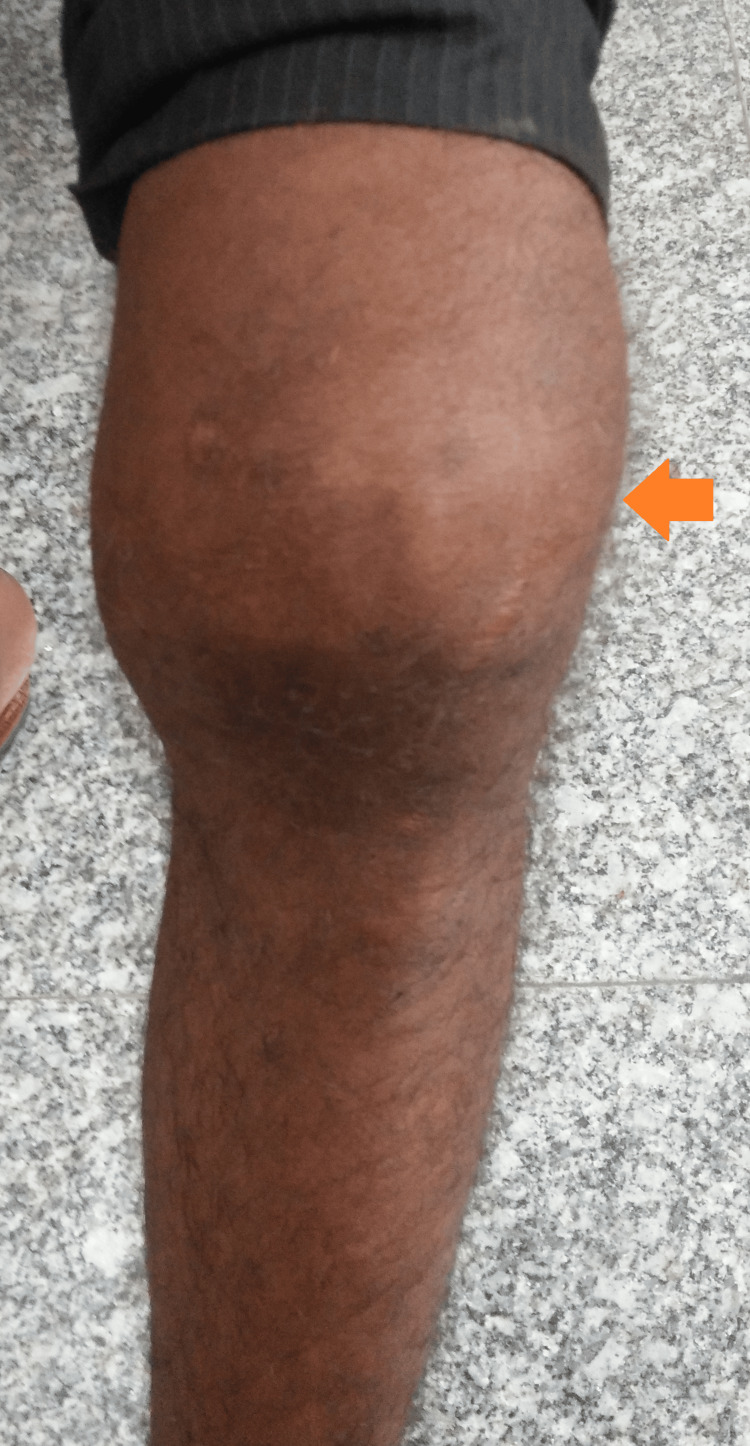
Gross image of the left knee showing diffuse swelling

Upon conducting a general examination, the patient presented as a slender individual with a body mass index of 17.6 kg/m². His hemodynamic status was stable. A localized examination of the left knee revealed a tender and diffusely swollen joint, with no signs of discharging sinuses or crepitus (Figure 1).

Active toe and ankle movements were observed, but there was limited mobility in the left knee joint, characterized by a positive patellar tap test and a deformity involving a 10-degree flexion, internal rotation, and external rotation. Moreover, the valgus and varus stress tests yielded negative results. The skin around the affected area exhibited erythema and an elevated temperature, although there were no dilated veins. Muscle wasting in the quadriceps of the left side was apparent, but there was no sensory loss. The right knee joint appeared normal. Importantly, there were no signs of jaundice, edema, lymph node enlargement, clubbing of the fingers, cyanosis, or pallor. Additionally, examinations related to vascular, pulmonary, abdominal, and neurological functions did not reveal any noteworthy findings.

Considering the possibility that the patient might have a pyogenic abscess, with alternative diagnoses in mind such as bone tumors, tuberculous osteomyelitis, and fungal osteomyelitis, a comprehensive laboratory and radiometric assessment plan was formulated.

**Figure 2:**
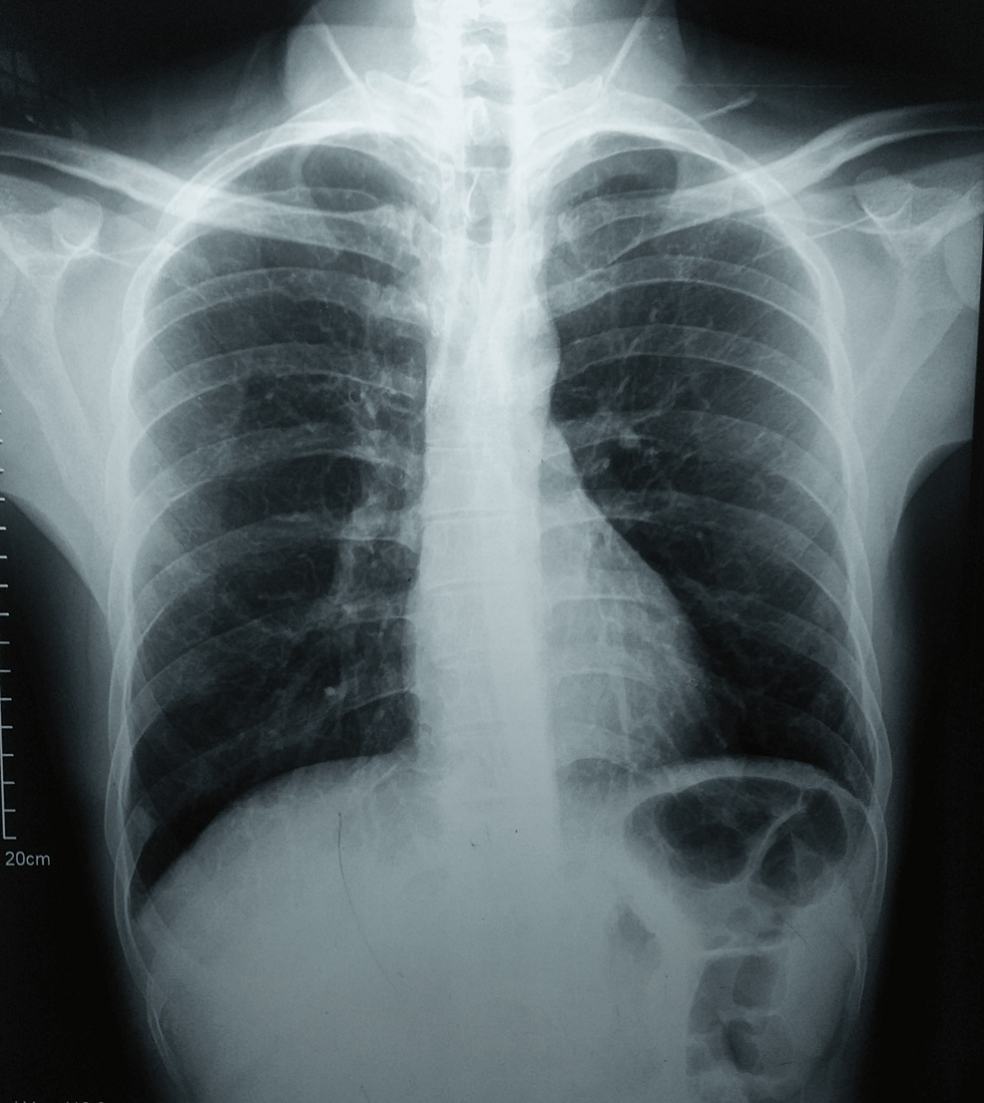
A plain chest radiograph not suggestive of pulmonary involvement

The results of the blood tests revealed some noteworthy findings, including an elevated erythrocyte sedimentation rate of 71 mm/hour (reference range ≤15 mm/hour) and a hemoglobin level of 11.8 g/dL. The rest of the tests, including HIV (I and II), hepatitis panel (A, B, and C), and rheumatoid factor tests, all returned negative results. However, the Mantoux test exhibited a strong positive reaction, measuring 25 mm. Furthermore, a plain chest radiograph did not indicate any signs of lung involvement (Figure 2).

**Figure 3:**
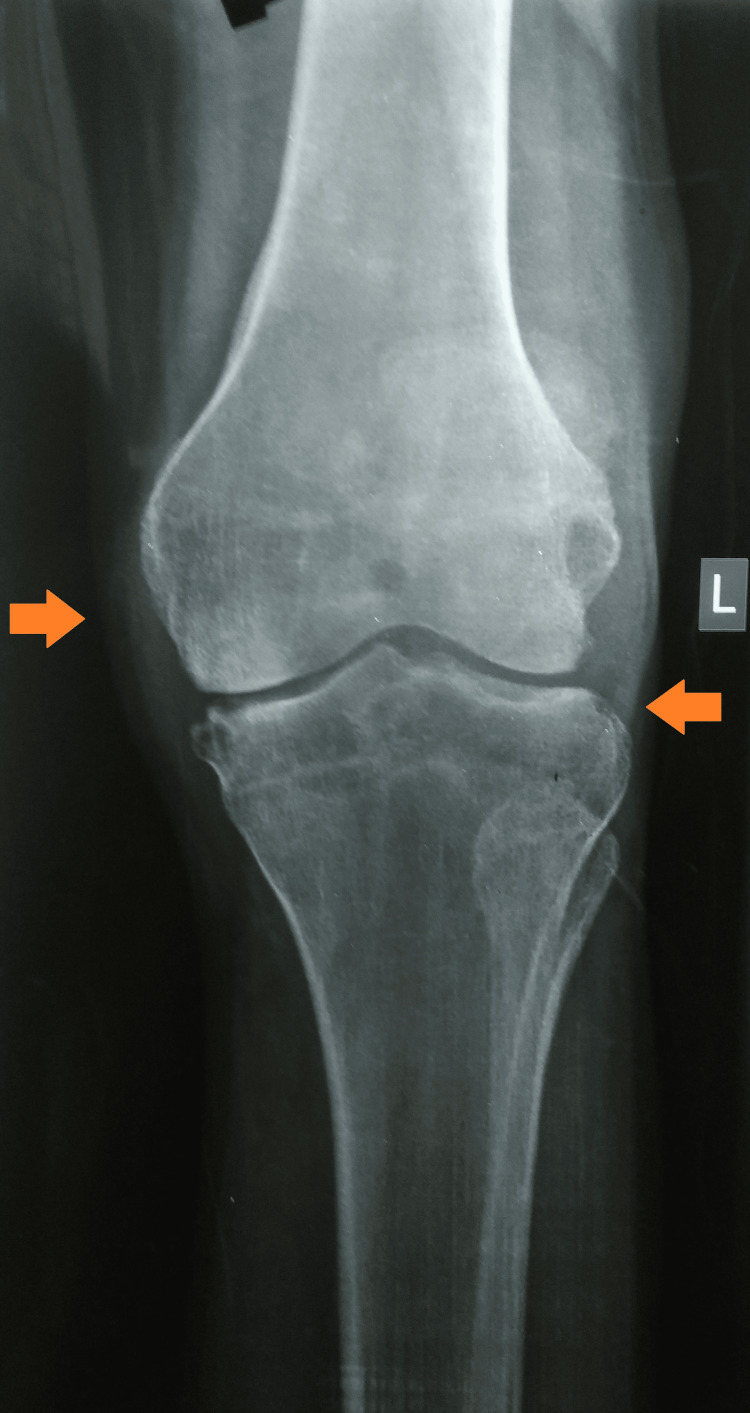
A plain radiograph of the left knee (AP view) was suggestive of knee joint involvement AP: Anteroposterior

**Figure 4:**
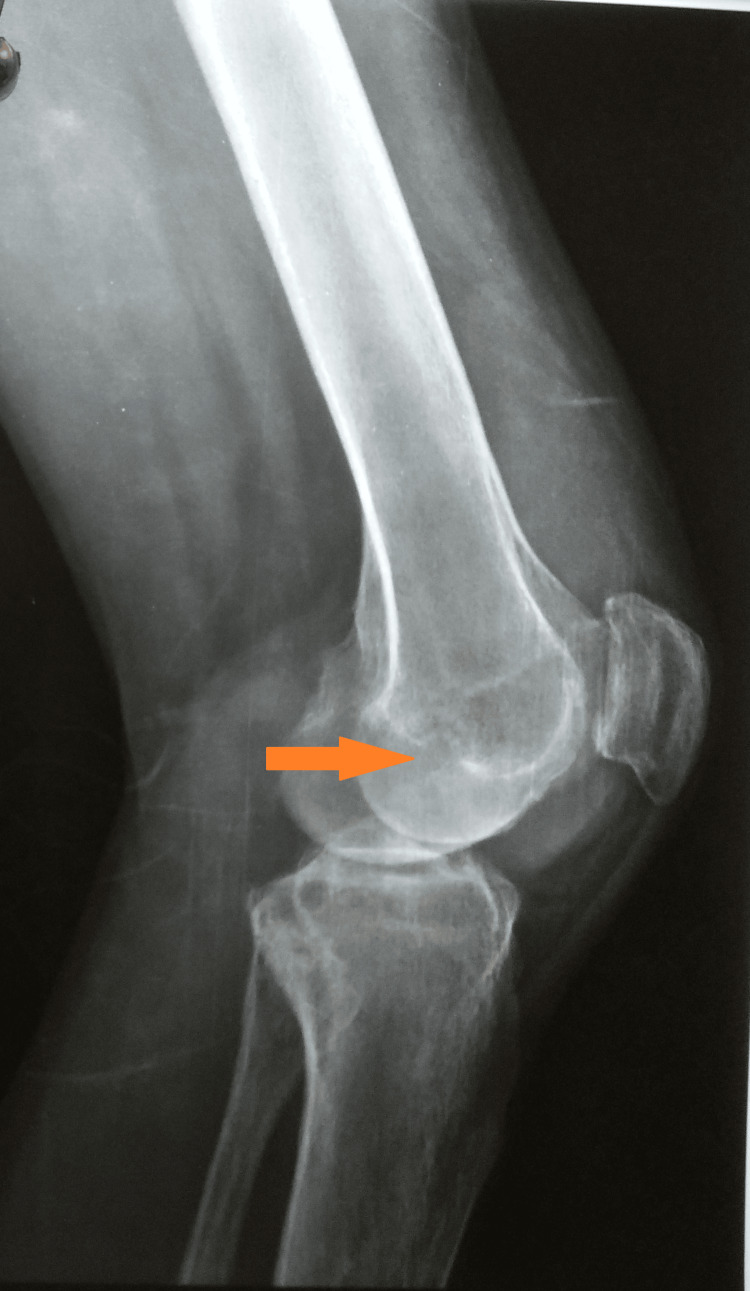
Plain radiograph of the left knee (lateral view) suggestive of tibial involvement

A plain radiograph of the affected knee showed peri-articular osteopenia, patella-femoral arthritis, and a lytic lesion in the medial condyle of the tibia (Figures 3, 4).

**Figure 5:**
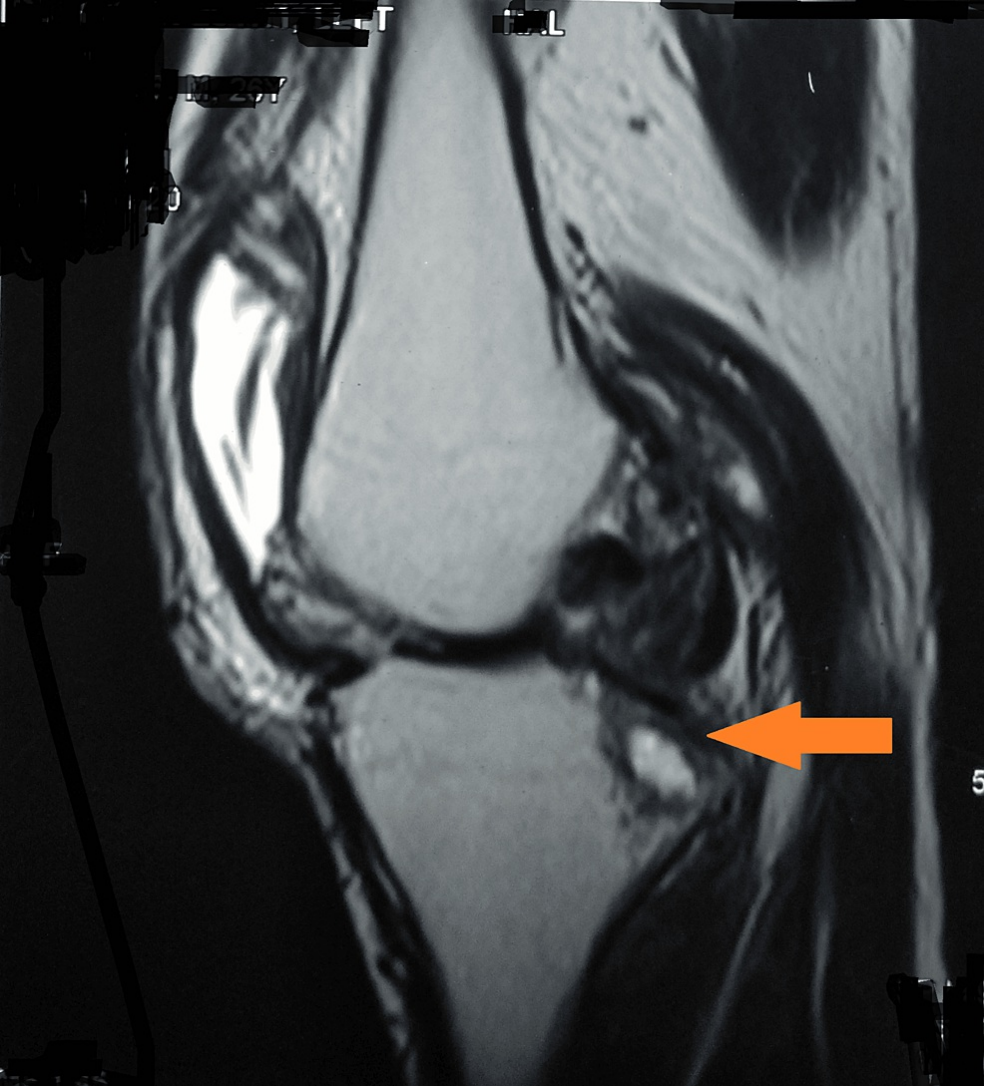
MRI of the left knee joint showing involvement of the knee joint MRI: magnetic resonance imaging

**Figure 6:**
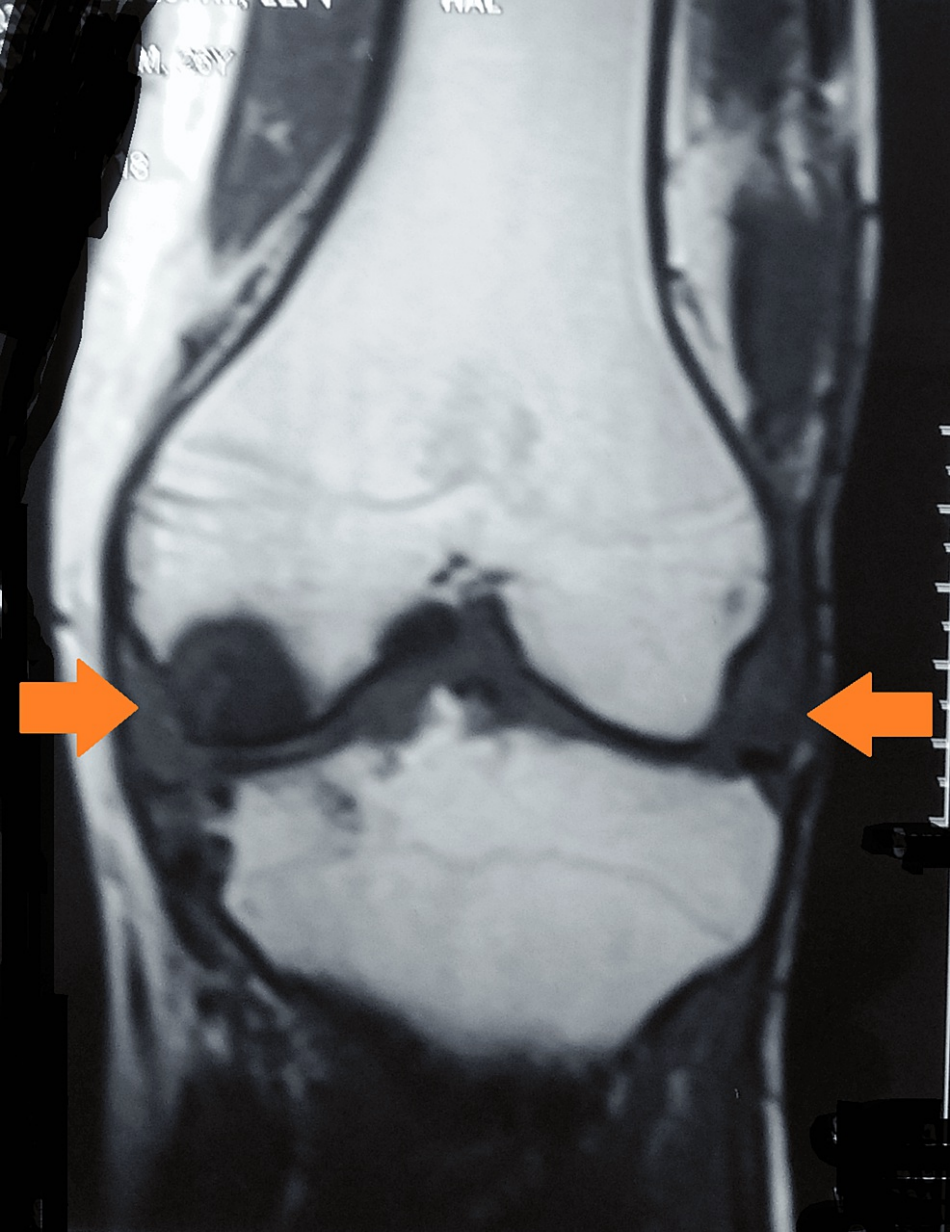
MRI of the left knee joint suggestive of knee joint involvement MRI: magnetic resonance imaging

Magnetic resonance imaging of the left knee joint showed an increased proton density fat saturation within the anteromedial and posteromedial bundles of anterior cruciate ligament fibers, suggesting myxoid degeneration. Mild diffuse edema was noted in the intercondylar fossa. Small tibio-femoral and patellar osteophytes were seen. Moderate to gross knee joint effusion was seen with synovial thickening in the suprapatellar bursa. A Grade II tear was seen in the posterior horn of the medial meniscus. Grade-II chondromalacia of patella changes were seen along the lateral patellar facet. Hyperintensity was noted as involving the medial condyle of the femur and medial tibia with adjacent soft tissue swelling and a Grade-I tear involving the lateral collateral ligament with bony erosion (Figures 5, 6).

Diagnostic arthroscopy and synovectomy with debridement were done under spinal anesthesia, and samples were sent for histopathology, a cartridge-based nucleic acid amplification test (CBNAAT), Ziehl-Neelsen staining for acid-fast bacilli (AFB), a line-probe assay, and culture. Arthroscopy revealed a hypertrophy of synovial tissue with a degenerated medial and lateral meniscus visible only at the meniscocapsular junction. There was detection of *Mycobacterium tuberculosis *(medium) with resistance to rifampicin on CBNAAT. The same was confirmed by histopathology reports suggestive of epitheloid granulomas in a necrotic background with Langhans giant cells and lymphocytic infiltrates. However, the rest of the tests were unremarkable.

A conclusive diagnosis was reached, indicating primary extrapulmonary rifampicin mono-resistant TB affecting solely the left knee joint, with no signs of pulmonary involvement. Subsequently, a pretreatment assessment was scheduled to commence the standard treatment regimen as outlined in the national guidelines [5]. Given the uneventful nature of his pretreatment evaluation, the decision was made to initiate an all-oral longer regimen, as detailed in Table 1.

**Table 1:** Anti-tubercular chemotherapy all-oral longer regimen per his weight OD: once daily

Drug	Route of administration	Dose	Duration
Bedaquiline	Per oral	400 mg X OD followed by 200 mg alternate day	2 weeks and then 22 weeks
Linezolid	Per oral	600 mg X OD, followed by 300 mg X OD	6 months and then 12 months
Moxifloxacin (high dose)	Per oral	800 mg X OD	540 days
Cycloserine	Per oral	750 mg X OD	540 days
Clofazimine	Per oral	100 mg X OD	540 days
Pyridoxine	Per oral	100 mg X OD	540 days

**Figure 7:**
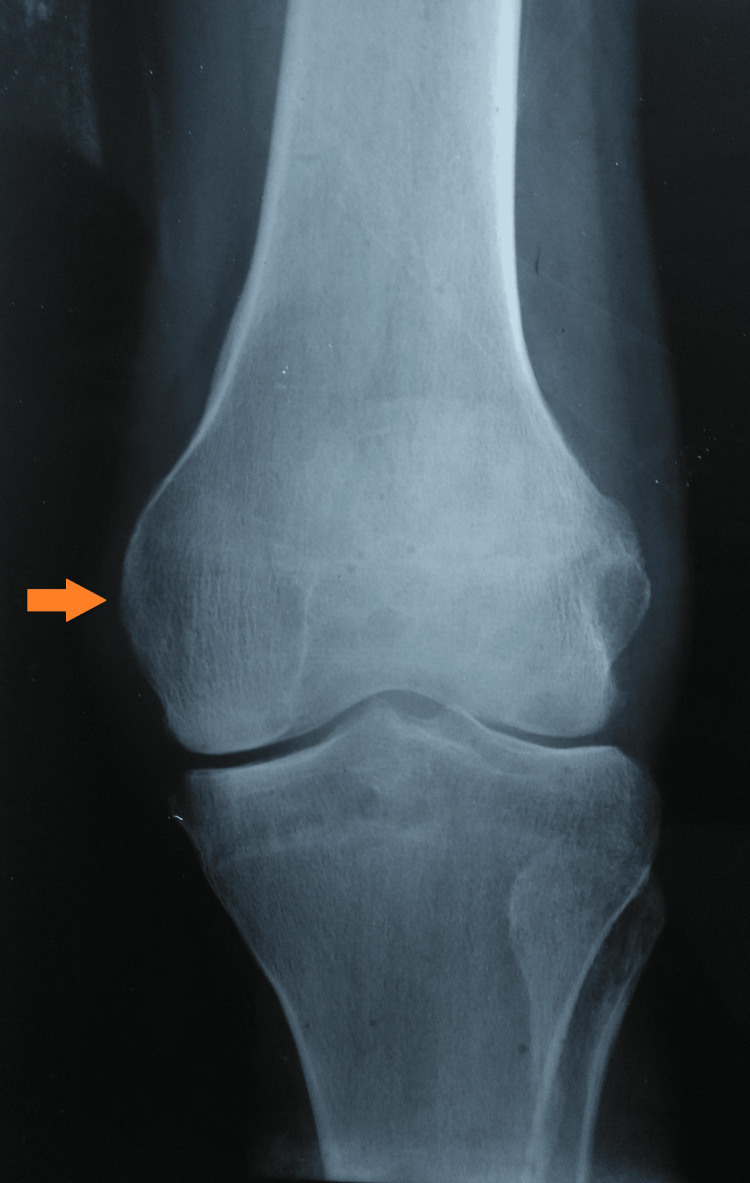
Plain radiograph of the left knee (AP view) showing no flaring-up of infection at the nine-month follow-up AP: anteroposterior

**Figure 8:**
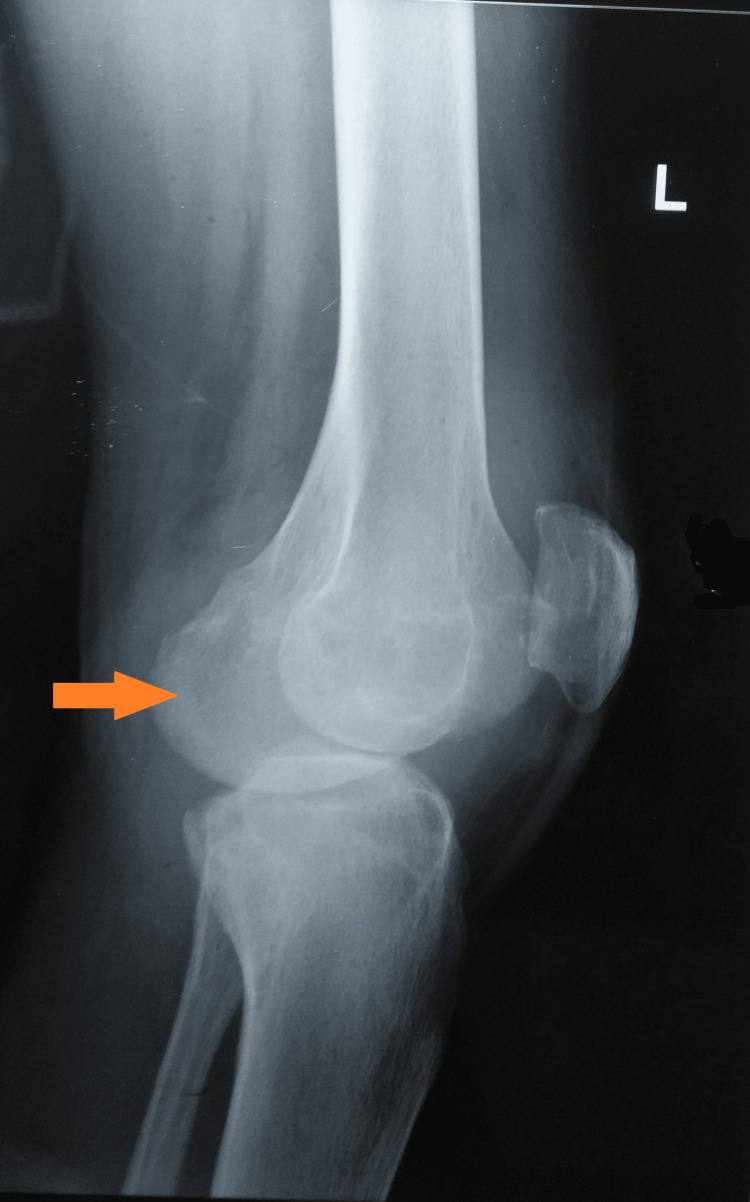
Plain radiograph (lateral view) of the left knee at the nine-month follow-up

He exhibited good tolerance for his anti-tubercular medications, experiencing no significant pharmacological side effects. Notably, there was a noticeable decrease in his symptoms. Furthermore, a follow-up knee radiograph conducted nine months later did not indicate any resurgence of the infection (Figures 7, 8).

As part of his ongoing care, a daily calcium tablet (500 mg) was incorporated into his regimen. Additionally, he received counseling regarding the importance of maintaining a healthy and hygienic life, along with strict adherence to his treatment plan. In accordance with his preference, he was referred to his hometown.

## Discussion

TB is caused by inhaling infected *Mycobacterium tuberculosis* through aerosols [6]. Post-1990, there was a notable increase in drug-resistant tuberculosis cases, posing a significant threat to TB control and elimination programs [7].

Musculoskeletal TB comprises approximately 3% of all TB cases [8]. Moreover, it makes up around 10-35% of all extrapulmonary TB cases [8]. This type of TB is most frequently observed in the spine (40%), followed by the hip (25%), and knee (8%) [8]. Instances of primary drug resistance in the absence of any prior history are infrequently documented [9]. It is exceedingly rare to encounter an extrapulmonary location such as the knee joint affected by a rifampicin mono-resistant strain of bacteria. A thorough review of the scientific literature has shown that there have been no reported cases of such a nature in males, particularly in the absence of any prior TB history.

Diagnosing such cases is challenging primarily because clinical and radiological characteristics lack specificity, constitutional symptoms of TB are frequently absent, primary care physicians may lack awareness and training, leading to delayed diagnosis, and the disease itself tends to have a low bacterial burden [10].

Typically, tests such as chest X-ray, the Mantoux test, and the interferon gamma-release assay offer a reasonably high level of sensitivity, which can be valuable for clinicians [10]. However, the gold standard for initiating treatment remains the isolation of AFB through Ziehl and Neelsen stain microscopy and bacteria culture [10]. Nevertheless, the challenge arises when there is an absence of *Mycobacterium tuberculosis* growth in culture, coupled with the paucibacillary nature of the disease, leading to potential misdiagnosis or a diagnostic delay [10]. In such cases, histopathology (with an 80% sensitivity rate) and a CBNAAT assume a pivotal role in determining the correct diagnosis [10].

The primary approach to treatment involves a conservative strategy utilizing anti-tubercular chemotherapy [10]. National programs provide established algorithms as guidelines for managing such cases [5]. Anti-tubercular drugs are known to cause adverse drug reactions; therefore, it is imperative to follow up with patients regularly. This would ultimately impact the treatment outcomes, as severe adverse drug reactions could be life-threatening. Surgical intervention is seldom warranted, typically considered only in instances of extensive disease or when there is a risk of disease reactivation [10].

There is a paucity of data on the rifampicin mono-resistant extrapulmonary involvement of the knee. A detailed PubMed search of the literature on the topic reveals no such presentation in the male gender. A similar case was presented in a young Indian female [11]. However, the present case differs from it in gender, history of trauma, and absence of discharging sinuses. Besides, one more case was published as an MDR TB case in a male, but that case had multiple issues such as the diagnosis, which was different from the claims made, the absence of an image, and the obscurity of the journal [12].

Additional delays in diagnosis could lead to dire complications [10]. Consequently, it is crucial for all such cases to be promptly initiated on treatment guided by drug-susceptibility testing. Given the scarcity of data concerning this condition, it's important to note that this case represents only a single instance. Gathering data from larger medical centers may prove valuable in refining prevailing guidelines, enabling a more directed approach to manage rifampicin mono-resistant knee joint TB.

## Conclusions

A case involving an Indian male is presented, who reported left knee swelling and pain. Given the absence of a TB history, arriving at a diagnosis proved to be a significant challenge. Nevertheless, through histopathological examination, a CBNAAT, and radiometric techniques, a diagnosis was established. Subsequently, he underwent a pretreatment evaluation following national guidelines and commenced an all-oral extended anti-tubercular regimen. There was noticeable development during the nine-month follow-up; however, he was strongly advised to complete the full 18-month course. It is imperative to stress the importance of disseminating information regarding such clinical presentations, as a lack of awareness and insufficient training could lead to unfavorable or fatal outcomes.
